# Utility of Progression Rate Since Symptom Onset in Predicting Subsequent Survival in Progressive Supranuclear Palsy

**DOI:** 10.1002/mds.70125

**Published:** 2025-11-17

**Authors:** Tao Xie, Lawrence I. Golbe

**Affiliations:** ^1^ Department of Neurology University of Chicago Medicine Chicago Illinois USA; ^2^ Department of Neurology Rutgers Robert Wood Johnson Medical School New Brunswick New Jersey USA

The search for markers of progressive supranuclear palsy (PSP) progression is important to neuroprotective clinical trial design and for patient counseling and disease management. We were therefore disappointed by the outcome of the excellent study by Quattrone et al,^1^ “Brain Atrophy Does Not Predict Clinical Progression in Progressive Supranuclear Palsy.” The authors found that neither clinical severity nor regional magnetic resonance imaging volumes predicted individual 12‐month trajectories in patients with PSP‐Richardson's syndrome.

That and most other attempts to model 12‐month PSP progression have used only baseline variables, but we have assessed the contribution of onset‐to‐baseline progression rates. First, in a pathologically verified PSP cohort, we found an association between the time from symptom onset to the first examination showing downgaze palsy (ie, scores >0 on PSP Rating Scale item 15) and overall survival duration.^2^ Next, via logistic regression in a prospective PSP cohort, we found that survival was associated with onset age and the progression rate of downgaze palsy as reflected by downgaze palsy severity and years since clinical onset.^3^ This conclusion was further validated in a much larger independent PSP cohort.^4^ We also similarly calculated a progression rate for other aspects of PSP, finding that the progression rate for dysphagia for liquids (PSPRS item 13) was associated with survival duration as well.^4^, ^5^

We therefore suggest that models to predict progression and survival in PSP include not only baseline findings but also progression rate of downgaze impairment and dysphagia from onset to baseline. As more compounds become candidates for advanced‐phase trials, innovations in trial design using such predictive models could reduce costs and improve statistical power to detect efficacy with fewer experimental subjects or shorter timelines.^6^

## Author Roles

(1) Research Project: A. Conception, B. Organization, C. Execution; (2) Statistical Analysis: A. Design, B. Execution, C. Review and Critique; (3) Manuscript: A. Writing of the First Draft, B. Review and Critique.

T.X.: 1A, 1B, 1C, 3A, 3B.

L.I.G.: 1A, 1B, 1C, 3B.

## Full Financial Disclosures for the Preceding 12 Months

TX: Stock Ownership in medically‐related fields: None. Intellectual Property Rights: None.

Consultancies: CVS Caremark. Expert Testimony: None. Advisory Boards: None. Employment: The University of Chicago Medicine. Partnerships: None. Inventions: None. Contracts: None. Honoraria: Parkinson's Foundation, Ono Pharmaceutical Co., Ltd, CVS Caremark. Royalties: None. Patents: None. Grants: The Michael J. Fox Foundation for Parkinson's Research, American Parkinson's Disease Association, and NIH. Other: None. L.I.G.: Stock Ownership in medically‐related fields: None. Intellectual Property Rights: None. Consultancies: AI Therapeutics, Amylyx, Aprinoia, Ferrer, Mitochon, NextCure/Alzprotect, and Woolsey. Expert Testimony: None. Advisory Boards: Amylyx, CurePSP (New York nonprofit), Rossy Center (Toronto nonprofit). Employment: None. Partnerships: None. Inventions: Share of licensing fees for the PSP Rating Scale and associated translations and instructional material, all via Rutgers University. Contracts: None. Honoraria: None. Royalties: Rutgers University Press (New Brunswick, NJ) for *A Clinician's Guide to PSP* (2019). Patents: None. Grants: None. Other: Travel expenses related to volunteer work with CurePSP, Inc. (New York, NY) as Scientific Advisory Board Chair, Chief Clinical Officer, and Board of Directors member.

## Data Availability

Data sharing not applicable—no new data generated.
